# The left ureterocele and stone of calyceal diverticulum in the patient with bilateral incomplete duplex kidneys managed by flexible ureteroscopy: a case report and literature review

**DOI:** 10.1186/s12894-020-00604-7

**Published:** 2020-03-30

**Authors:** Yang Pan, Gang Chen, Han Chen, Yunxiao Zhu, Hualin Chen

**Affiliations:** Department of Urology, The First Affiliated Hospital of Chongqing Medical University, No. 1 Road Youyi, Yuzhong District, Chongqing, China

**Keywords:** Ureterocele, Stone, Calyceal diverticulum, Duplex kidneys, Flexible ureteroscopy, Case report

## Abstract

**Background:**

Duplex kidneys are one of the most common renal congenital abnormalities, mostly asymptomatic and of no clinical significance. There are little reports about the left ureterocele and stone of calyceal diverticulum in patients with bilateral incomplete duplex kidneys managed by flexible ureteroscopy.

**Case presentation:**

A 69-year-old Chinese woman was presented with left waist pain for 1 month. A preoperative computed tomography (CT) scan and intravenous pyelogram revealed the left ureterocele which located in the left ureterovesical junction, and stone of calyceal diverticulum which located in the upper kidney of left incomplete duplex kidneys. The ureterocele was confirmed in view of ureteroscopy and the holmium laser was used for the resection of ureterocele. It took us a lot of efforts to find out the stone because of diverticular neck stenosis. Fortunately, when diverticular neck stenosis was incised internally by holmium laser, the stone was discovered clearly and removed using the holmium laser and nitinol stone basket through flexible ureteroscopy. A double-J ureteral stent was inserted and remained in place for 1 month. The symptom disappeared postoperatively and no complications were developed during the placement of the stent. There were no stone residents observed on CT scan before removing the ureteral stent 1 month later.

**Conclusions:**

Flexible ureteroscopy with holmium laser is feasible to manage the ureterocele and calyceal diverticulum stones in patients with bilateral incomplete duplex kidneys in one operation.

## Background

Duplex kidneys are common congenital malformations of urogenital system, with an incidence of approximately 1–3% [1, 2]. Duplex kidneys can be categorized as complete or incomplete according to ureteral morphology [3]. Meanwhile, such a duplication also can be bilateral or unilateral, and some studies have reported that only 0.3% of the patients are detected bilateral duplex kidneys by excretory urography [4, 5].

Most patients with duplex kidneys have no significant clinical symptoms. But some complications like urolithiasis and vesicoureteral reflux (VUR) are relevant to duplex kidneys [5]. Iatrogenic ureteral injury is a possible risk during many urogynecology surgeries, and it’s more likely to occur when the ureter exists anatomical varieties [6].

Percutaneous nephrolithotomy (PCNL), retrograde intrarenal surgery (RIRS) and extracorporeal shock wave lithotripsy (SWL) are now the standard treatment modalities for the majority of upper tract stone diseases [7]. SWL shows a relatively lower stone-free rate (SFR), while PCNL represents a higher risk of bleeding [8]. Flexible ureteroscope can reach the pelvicalyceal system easily, and it is beneficial to the improvement of SFR. As a result, RIRS combined with holmium laser lithotripsy has become a less-invasive and preferred procedure for the management of < 20 mm upper urinary tract stones on the basis of its high SFR and acceptable complication rates [9, 10].

We report a clinical case of the left ureterocele and stone of calyceal diverticulum in the patient with bilateral incomplete duplex kidneys managed by flexible ureteroscopy. At the same time, we conduct some literature reviews and provide some suggestions on the treatment of the ureterocele and stones of calyceal diverticulum in patients with duplex kidneys.

## Case presentation

**Fig. 1:**
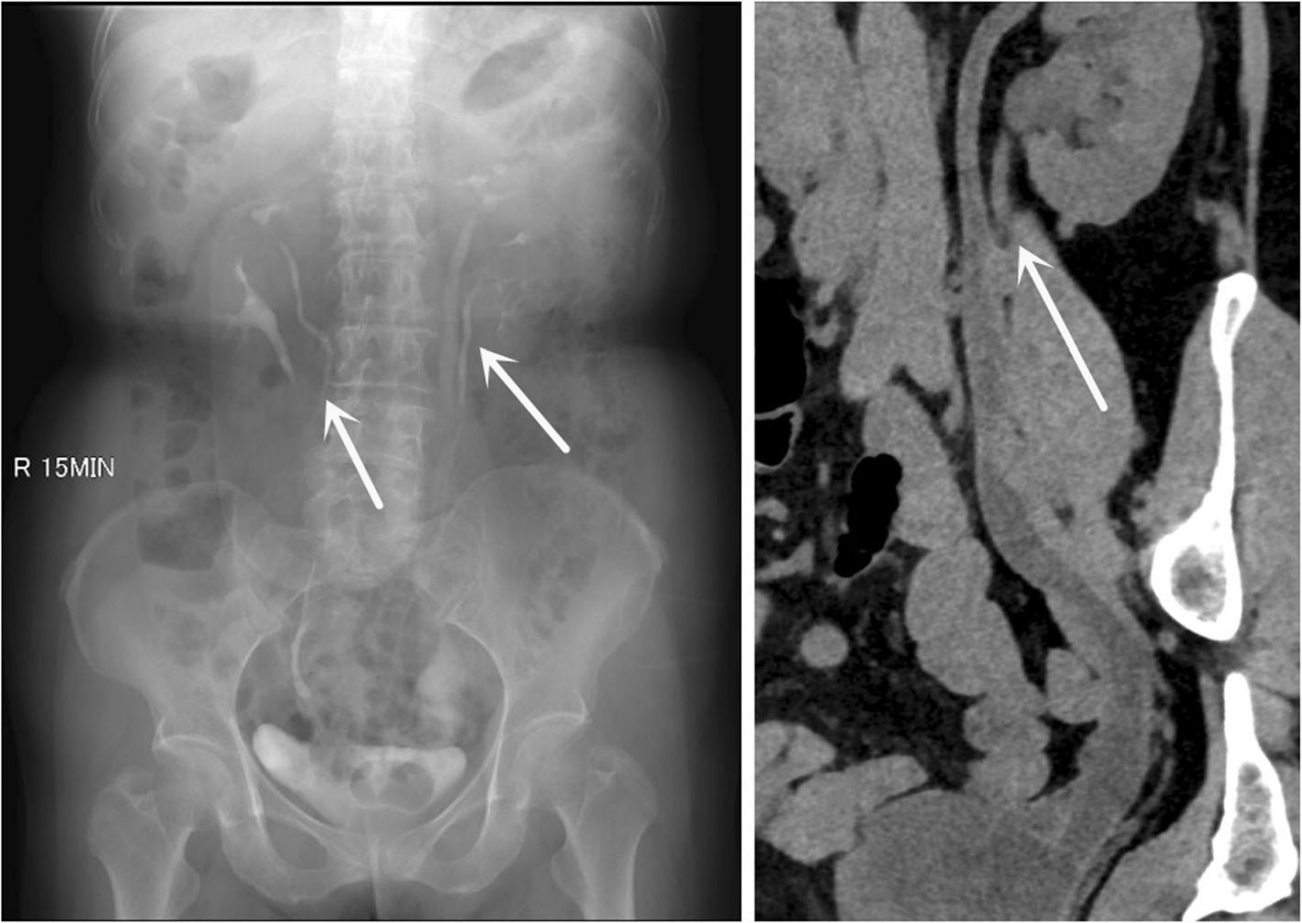
Intravenous pyelogram and CT scan revealed bilateral incomplete duplex kidney and ureter

**Fig. 2:**
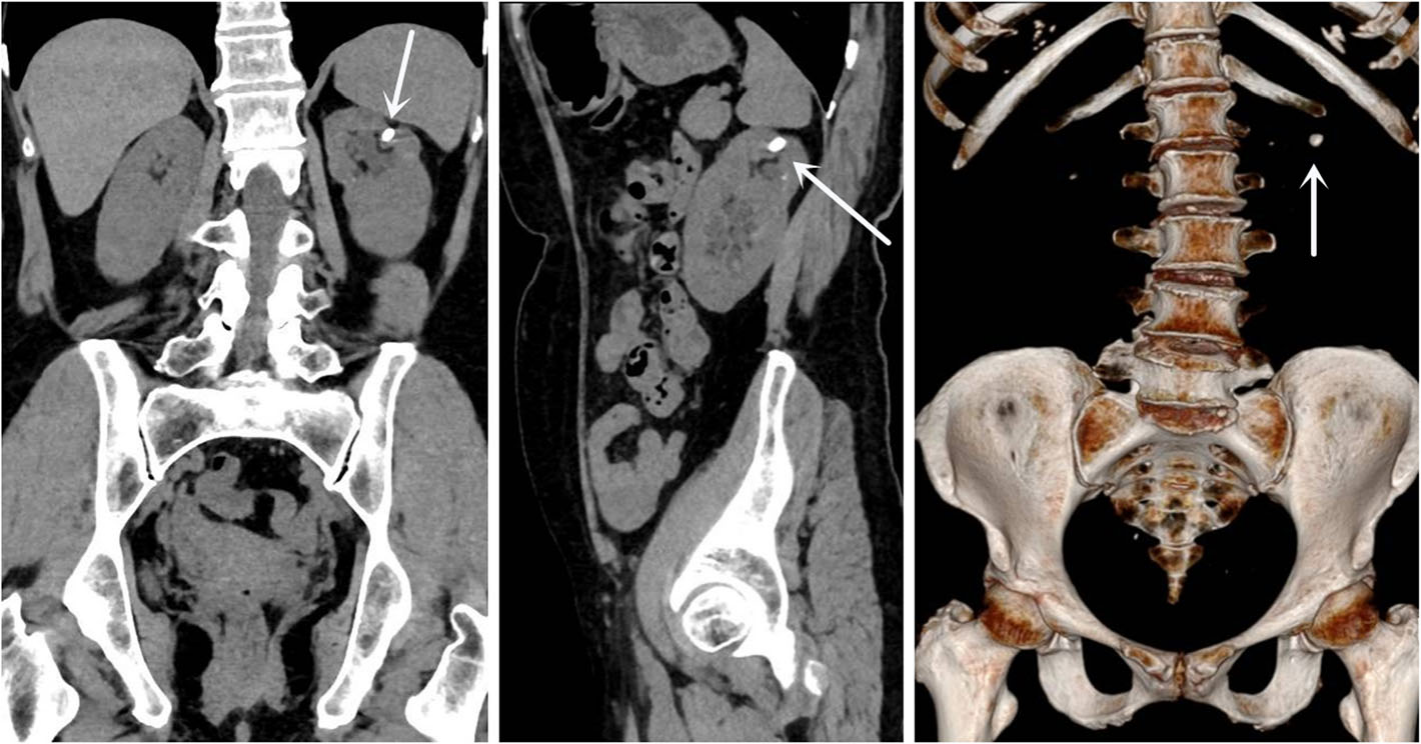
The stone of calyceal diverticulum was located in the upper kidney of left incomplete duplex kidneys by CT scan

**Fig. 3:**
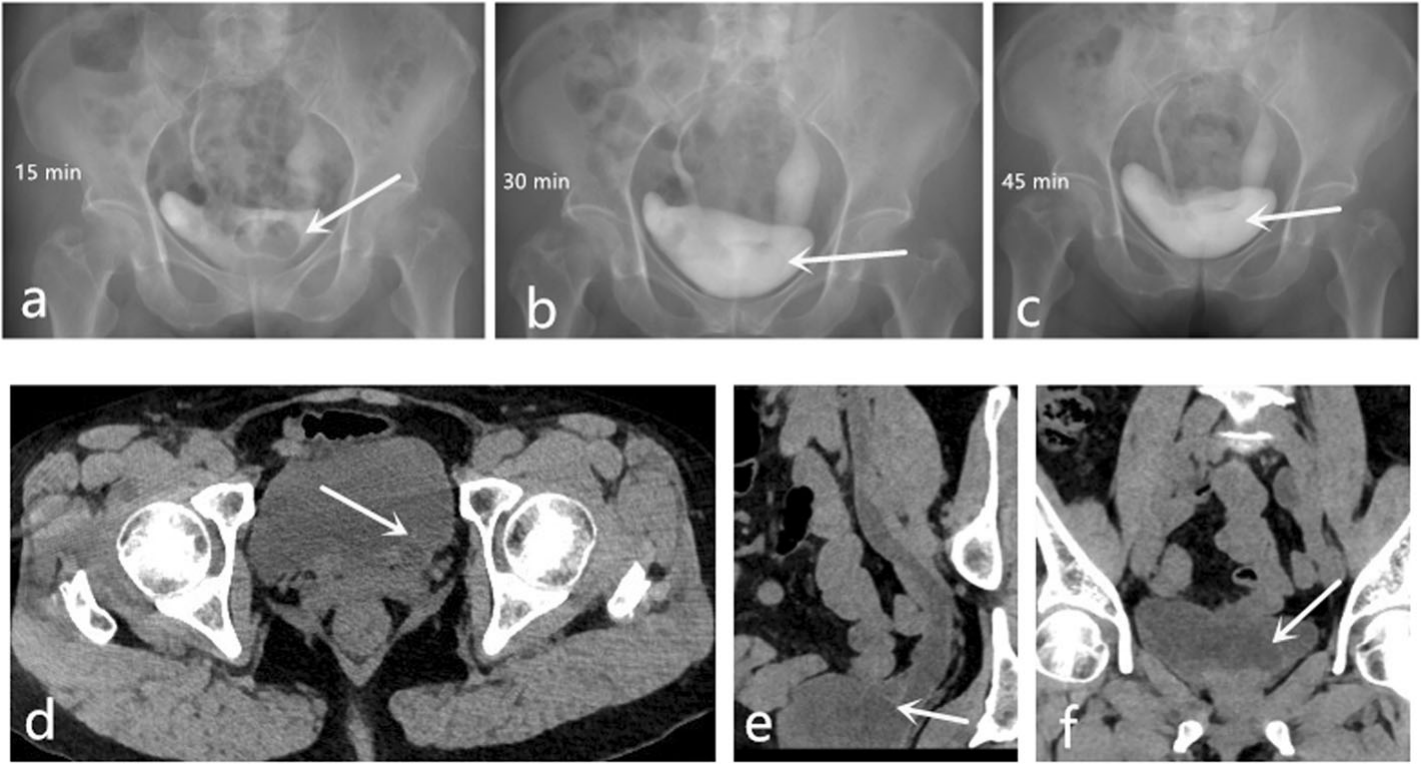
Intravenous pyelogram at 15 (**a**), 30 (**b**), 45 (**c**) minutes respectively after injecting contrast medium revealed a ureterocele. CT scan (**d**, **e**, **f**) revealed that a ureterocele was located in the left ureterovesical junction

A 69-year-old Chinese woman was presented with left waist pain for 1 month. She had no fever or other pain. There were no abnormal results in the blood routine examination, renal function, and urine routine examination. And the result of urine culture was negative. A preoperative computerized tomography (CT) scan and intravenous pyelogram (IVP) revealed bilateral incomplete duplex kidney and ureter (Fig. 1). The stone of calyceal diverticulum was located in the upper kidney of left incomplete duplex kidneys by CT scan (Fig. 2). In the meanwhile, IVP and CT scan revealed that a ureterocele was located in the left ureterovesical junction (Fig. 3).

The patient had hypertension and type 2 diabetes mellitus for 5 years. The risks of surgery and general anesthesia were relatively higher. The diameter of the stone in the calyceal diverticulum was 12 mm by CT scan and it’s difficult for the stone to pass out spontaneously. The patient and her family wished to remove the stone because they were extremely worried about stone-related complications. Moreover, they wouldn’t like to perform second operation and anesthesia due to relatively higher risks. Therefore, we planned to make an attempt to remove the ureterocele and calyceal diverticulum stone in one operation.

The contraindications for surgery in such cases mainly included untreated urinary tract infection, severe urinary tract stricture, and anesthetic contraindications like cardiopulmonary dysfunction. Relevant examinations such as lung-function testing and Holter electrocardiogram were conducted. We also paid special attention to the results of urine routine and urine culture. When we made sure all these examination results were normal, we decided to perform the surgery.

**Fig. 4:**
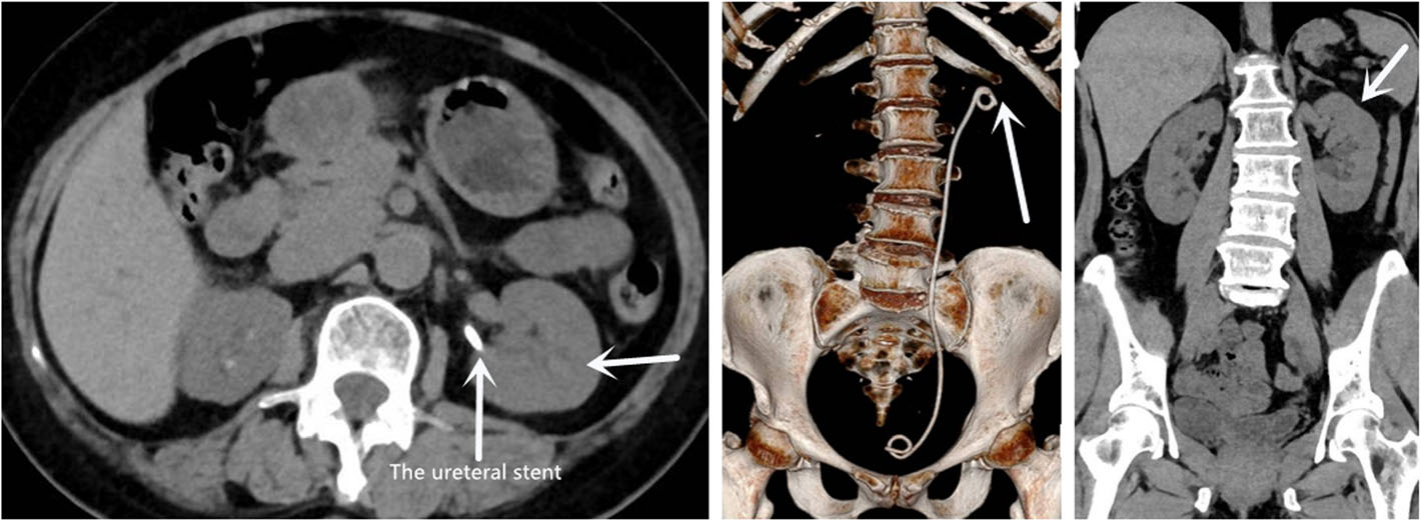
There were no stone residents observed on CT scan 1 month later postoperatively

Flexible ureteroscopy with the holmium laser was conducted for solving the ureterocele and stone of calyceal diverticulum. To begin with, the ureterocele was confirmed in view of ureteroscopy and the holmium laser was used for the resection of ureterocele. The ureterocele resection surgery was completed in 10 min and no complications like bleeding occurred intraoperatively. Then, we made an attempt to remove the stone of calyceal diverticulum. During the operation, we found the calyx neck of calyceal diverticulum where the stone was located had obvious stenosis. It took us a lot of effort to find out the stone because of calyx neck stenosis. Fortunately, the stone was discovered when calyx neck stenosis was incised internally by holmium laser. After dilatation of the narrow calyx neck, a 200-μm holmium laser through flexible ureteroscope was used to make the stone fragmented gradually and carefully. The parameter of the holmium laser was set at a power of 0.8 J and a pulse frequency of 10 Hz. Higher power and frequency might cause renal damage and hemorrhage because the calyceal diverticulum stone was near the edge of the kidney and the renal cortex was thin by CT scan. In order to prevent the formation of ureteral steinstrasse postoperatively, a nitinol stone basket was used to remove large stone fragments as soon as possible, and the smaller powdered fragments were flushed out of the diverticulum using an automated irrigation pump. At last, the pelvicalyceal system was examined once again to ensure no large remaining stones. The overall surgical time was controlled in 60 min for the purpose of preventing postoperative infections. A double-J ureteral stent was inserted and remained in place for 1 month. The symptom of left lumbar back pain disappeared post the operation and no complications were developed during the placement of the stent. There were no stone residents observed by CT scan before removing the ureteral stent 1 month later (Fig. 4).

## Discussion and conclusions

Duplex kidneys refer to a kind of renal congenital anatomical malformations in which the renal unit is made up of two separate pelvicalyceal systems [11, 12]. The renal vessels, collecting system and ureter of duplex kidneys are respectively separated. The incidence of duplicate kidney and ureter is about 1~3% [4]. A few malformations can accompany ureteral ectopic opening or ureteral protrusion. Most patients with duplex renal and ureteral malformations have no specific clinical symptoms. It was often found in physical examination, or complicated with stone, hydronephrosis, infection and other symptoms.

At present, some studies have shown that ultrasound is a major imaging examination for the survey of duplex kidneys [13]. Ultrasound is extremely conducive to diagnose because of its simplicity and convenience. Furthermore, it is not radioactive to the patient. IVP and CT are also important imaging methods to diagnose the disease, moreover, the sensitivity of CT is better than the former two. According to the studies, the diagnostic coincidence rates of urinary system for B ultrasound, IVP and CT were 44.7, 60.0 and 100%, respectively. At the same time, the diagnostic coincidence rates of CT for hydronephrosis, calculi, ectopic ureteral orifice and ureterocele were also higher than those of B ultrasound and IVP.

Duplex kidney and ureter malformations can be divided into complete and incomplete according to ureteral morphology. The former refers to two independent pelvicalyceal systems derived from two ureteric buds of the mesonephric duct [14, 15]. Two resulting ureters have a respective fusion to the mesenchyme and cause isolated drainage of ipsilateral kidney. These two ureters were identified as lower or upper moiety according to the corresponding location. The opening position of ureter conforms to the Weigert–Meyer rule [16]. It means that the ureter from the upper moiety of the complete duplex kidney usually opens below and medial to the one from the lower moiety [2, 15].

Incomplete duplex kidney and ureter are derived from a premature division of the ureteral bud before fusing with the renal mesenchyme. Bifid moieties form at the proximal of ‘Y-shaped’ ureter, however, a confluence occurs at the distal of the ureter eventually. The confluence occurs in the upper and lower ureters respectively accounting for 25% and the middle ureter accounts for 50% [17].

Due to the unique anatomical abnormalities of duplex kidney and ureter malformations, it is easy to appear urine drainage [18]. The drainage can result in upper urinary tract obstruction, infection, calculi and other complications. The treatment of duplex renal and ureteral malformations combined with upper urinary calculi, is mainly based on the size of the calculi, location, degree of obstruction, whether combined with lumbago, hematuria, infection, kidney damage or not. If it is a small duplex kidney stone, without clinical symptoms, and don't cause hydronephrosis or renal function damage, it can be observed or conservative treatment.

Most patients with duplex kidneys have no significant symptoms, but complications are relatively frequent [19]. The complications of duplex kidneys can be classified as the upper moiety complications and lower moiety complications, and the former is significantly more common according to a few current literature [1, 4, 14]. Upper moiety complications are related to the ureteral ectopic insertion, usually complicated with a ureterocele or multi-cystic dysplastic moiety kidney. Males may occur a pelvic mass if urine gradually drains into an accessory sexual structure; Females can experience urinary incontinence when the inserting position is under the urethral sphincter [18, 20]. The most common upper moiety complication is urinary tract obstruction due to a ureterocele [4].

Lower moiety complications are mainly caused by the lateral shift of the ureters insertion into the bladder and consist of VUR, renal scarring and pelviureteric junction (PUJ) obstruction [18]. The most prevalent lower moiety complication is VUR and it can lead to recurrent urinary tract infections (UTIs) which may have a permanent impairment on renal function [21, 22]. Saddle reflux is a special complication unique to duplex kidneys and it means that the reflux occurs from one proximal end of the bifid ureter of incomplete duplication [4]. PUJ obstruction in duplex kidneys was found to be less than 1% according to current literature [23].

At present, a variety of minimally invasive surgeries have gradually replaced the traditional open surgery. If surgical treatment is required, clinicians can provide individualized minimally invasive treatment for patients according to the size, location, degree of hydronephrosis, renal function, patient willingness, operator experience and so on. Nowadays, the minimally invasive surgical methods for duplex kidneys with upper urinary stones mainly include ESWL, PCNL, Ureteroscope lithotripsy (URL), laparoscopic ureterolithotomy, etc. Although ESWL has the advantages of no anesthesia, simplicity, safety and small trauma in the treatment of duplex kidneys stones, however, the rate of re-treatment is as high as 58.6% [24]. PCNL, on the other hand, is relatively minimally invasive, with high lithotripsy efficiency and rapid postoperative recovery. But the risk of bleeding and injury to surrounding organs should not be ignored. Laparoscopic ureterolithotomy is suitable for patients with large upper ureteral calculi when other minimally invasive methods don’t work. It can replace open surgery; however, relatively large trauma and ureteral stenosis are easy to occur. The ureteroscopy can only handle middle and lower ureteral calculi, while the flexible ureteroscopy lithotripsy can handle upper ureteral and renal calculi with great advantages. To some extent, it can overcome the poor effect of ESWL lithotripsy, the need for multiple treatments, puncture trauma and high risk of bleeding of PCNL, and it is also a better choice for teenagers [25].

Combined with our surgical experience, we choose flexible ureteroscopy with holmium laser for the management of upper urinary tract stones in patients with duplex kidney and ureter malformations. And we conclude the following experience:

First, due to the anatomic abnormality of duplex kidney and ureter malformations, choosing a small size ureteroscope as far as possible to reduce the difficulty to enter ureteral lumen and the damage to ureter wall while there exists ureteral stenosis. For upper ureteral calculi, ureteroscopy and holmium laser lithotripsy can be used firstly. After the calculi fall into the renal pelvis, flexible ureteroscopy can be utilized. This can shorten the time of using flexible ureteroscopy and reduce the consumption of machines. Although the image of the electronic flexible ureteroscope is clearer, the diameter is larger than the fibrous flexible ureteroscope. When choosing the electronic flexible ureteroscope, it may be difficult to enter the lateral half kidney with the calculi. Moreover, during the process of lithotripsy, the outflow from the ureteral catheter sheath is extremely slow, and the pressure in the renal pelvis is high, which increases the risk of urosepsis after surgery. As a result, the fibrous flexible ureteroscope is recommended.

Second, for patients with incomplete duplex kidney malformations, making sure that the ureteroscope gets to the double ureteral fusion position firstly. After identifying the bifurcation of two ureters, the specific location of the calculi can be determined according to the preoperative examination results. Then, the guidewire is placed in the side of the ureter with the calculi. It can also avoid unnecessary damage when entering the non-calculous ureter. Usually, the distal end of the ureteral catheter sheath should be placed to the ureteral fusion position, which can avoid the ureter bifurcation avulsion caused by the passive expansion of the sheath.

Third, for patients with complete duplicate renal and ureteral malformations, the position of calculi should be found combining with preoperative IVU or CTU in order to avoid the embarrassing situation that the calculi can’t be found. And the ureteral openings should be found according to Weigert-Meyer’s rule. The upper kidney and ureter are usually lean inward and downward, while the lower kidney and ureter are usually located outward.

Forth, for a small number of patients with incomplete duplicate kidney and ureter malformations, the stricture could exist at the bifurcation of the double ureter. As a result, the internal incision and balloon dilatation of ureteral stricture can be conducted through ureteroscopy firstly. Then, flexible ureteroscopy lithotripsy can be performed, and two double J ureteral stents can try to be retained to avoid ureteral stricture postoperatively.

Fifth, the angle between the repeated ureter and the corresponding lower renal calyx should be carefully evaluated before the operation when the calculi are located in the lower renal calyx. PCNL can be considered in the case of the following conditions: 1) flexible ureteroscope can’t enter the lower renal calyx with an extremely small angle; 2) the ureteral catheter sheath can’t be placed because the ureter is narrow or distorted severely; 3) the calculi are too large with the diameter of greater than 2 cm.

Duplex kidneys and ureter are one kind of common urological malformations, which are often of no obvious symptoms. Whereas there are nearly half of patients with duplex kidneys who need to be managed because of all kinds of comorbidities. A ureterocele and VUR are considered as the most frequent upper moiety complication and lower moiety complication, respectively. While the patient has the ureterocele and bilateral incomplete duplex kidney and ureter combined with calyceal diverticulum stones, flexible ureteroscopy and holmium laser lithotripsy might be a feasible and effective choice.

The patient in our report had four special comorbidities including the left ureterocele, calyceal diverticulum stone, diverticular neck stenosis, and bilateral incomplete duplex kidney and ureter. It’s relatively rare that four comorbidities occurred in one patient. What’s more, we conducted a minimally invasive surgery for the resection of ureterocele, the dilatation of diverticular neck, and the removal of calyceal diverticulum stone. Therefore, the special features of our report were made up of the rare four coexisting diseases and one endoscopic operation for solving three problems.

In conclusion, it appears that flexible ureteroscopy and holmium laser is a feasible, effective and minimally invasive way for managing the ureterocele and stones of calyceal diverticulum in patients with bilateral incomplete duplex kidneys in one operation.

## Data Availability

The datasets used and/or analyzed during the current study are available from the corresponding author on reasonable request.

## Abbreviations

VUR: Vesicoureteral reflux
PCNL: Percutaneous nephrolithotomy
SWL: Extracorporeal shock wave lithotripsy
RIRS: Retrograde intrarenal surgery
SFR: Stone free rate
CT: Computerized tomography
IVP: Intravenous pyelogram
UTIs: Urinary tract infections
PUJ: Pelviureteric junction
URL: Ureteroscope lithotripsy

## References

[1] Hunziker M, Kutasy B, D'Asta F, Puri P (2012). Urinary tract anomalies associated with high grade primary vesicoureteral reflux. Pediatr Surg Int.

[2] Davda S, Vohra A (2013). Adult duplex kidneys: an important differential diagnosis in patients with abdominal cysts. JRSM Short Rep.

[3] Varlatzidou A, Zarokosta M, Nikou E, Theodoropoulos P, Kakaviatos D, Piperos T (2018). Complete unilateral ureteral duplication encountered during intersphincteric resection for low rectal cancer. J Surg Case Rep.

[4] Privett JT, Jeans WD, Roylance J (1976). The incidence and importance of renal duplication. Clin Radiol.

[5] Papageorgiou D, Kyriazanos I, Zoulamoglou M, Deskou E, Kalles V, Stamos N (2019). Incomplete bilateral duplication of the ureters identified during cytoreductive surgery for ovarian cancer: a case report. Int J Surg Case Rep.

[6] Selzman AA, Spirnak JP (1996). Iatrogenic ureteral injuries: a 20-year experience in treating 165 injuries. J Urol.

[7] Mehmet NM, Ender O (2015). Effect of urinary stone disease and its treatment on renal function. World J Nephrol.

[8] Akman T, Binbay M, Tekinarslan E, Ozkuvanci U, Kezer C, Erbin A (2011). Outcomes of percutaneous nephrolithotomy in patients with solitary kidneys: a single-center experience. Urology..

[9] Turk C, Petrik A, Sarica K, Seitz C, Skolarikos A, Straub M (2016). EAU guidelines on interventional treatment for urolithiasis. Eur Urol.

[10] Yang B, Ning H, Liu Z, Zhang Y, Yu C, Zhang X (2017). Safety and efficacy of flexible ureteroscopy in combination with holmium laser lithotripsy for the treatment of bilateral upper urinary tract calculi. Urol Int.

[11] Hartman GW, Hodson CJ (1969). The duplex kidney and related abnormalities. Clin Radiol.

[12] Horst M, Smith GH (2008). Pelvi-ureteric junction obstruction in duplex kidneys. BJU Int.

[13] Ladwig SH, Older RA, Foster WL, Korobkin M (1982). Computed tomographic diagnosis of the obstructed duplex kidney in adults. N C Med J.

[14] Whitten SM, Wilcox DT (2001). Duplex systems. Prenat Diagn.

[15] Malek RS, Kelalis PP, Stickler GB, Burke EC (1972). Observations on ureteral ectopy in children. J Urol.

[16] Lee KH, Gee HY, Shin JI (2017). Genetics of vesicoureteral reflux and congenital anomalies of the kidney and urinary tract. Investig Clin Urol.

[17] Geavlete P, Nita G, Georgescu D, Mirciulescu V (2001). Endoscopic classification and endourologic therapy in proximal incomplete ureteral duplication pathology. Eur Urol.

[18] Doery AJ, Ang E, Ditchfield MR (2015). Duplex kidney: not just a drooping lily. J Med Imaging Radiat Oncol.

[19] South Bedfordshire Practitioners’ Group (1990). Development of renal scars in children: missed opportunities in management. BMJ.

[20] Chertin B, Mohanan N, Farkas A, Puri P (2007). Endoscopic treatment of vesicoureteral reflux associated with ureterocele. J Urol.

[21] Stokland E, Jodal U, Sixt R, Swerkersson S, Hansson S (2007). Uncomplicated duplex kidney and DMSA scintigraphy in children with urinary tract infection. Pediatr Radiol.

[22] Thomas JC (2008). Vesicoureteral reflux and duplex systems. Adv Urol.

[23] Gonzalez F, Canning DA, Hyun G, Casale P (2006). Lower pole pelvi-ureteric junction obstruction in duplicated collecting systems. BJU Int.

[24] Tunc L, Tokgoz H, Tan MO, Kupeli B, Karaoglan U, Bozkirli I (2004). Stones in anomalous kidneys: results of treatment by shock wave lithotripsy in 150 patients. Int J Urol.

[25] Jurkiewicz B, Zabkowski T, Samotyjek J (2014). Ureterolithotripsy in a paediatric population: a single institution's experience. Urolithiasis..

